# Cloning of the *pks3* gene of *Aurantiochytrium limacinum* and functional study of the 3-ketoacyl-ACP reductase and dehydratase enzyme domains

**DOI:** 10.1371/journal.pone.0208853

**Published:** 2018-12-11

**Authors:** Zhu Liu, Xiaonan Zang, Xuexue Cao, Zhendong Wang, Chang Liu, Deguang Sun, Yalin Guo, Feng Zhang, Qin Yang, Pan Hou, Chunhong Pang

**Affiliations:** Key Laboratory of Marine Genetics and Breeding, Ministry of Education, Ocean University of China, Qingdao, People’s Republic of China; Universite Paris-Sud, FRANCE

## Abstract

*Aurantiochytrium limacinum* has received attention because of its abundance of polyunsaturated fatty acids (PUFAs), particularly docosahexaenoic acid (DHA). DHA is synthesized through the polyketide synthase (PKS) pathway in *A*. *limacinum*. The related enzymes of the PKS pathway are mainly expressed by three gene clusters, called *pks1*, *pks2* and *pks3*. In this study, the full-length *pks3* gene was obtained by polymerase chain reaction amplification and Genome Walking technology. Based on a domain analysis of the deduced amino acid sequence of the *pks3* gene, 3-ketoacyl-ACP reductase (KR) and dehydratase (DH) enzyme domains were identified. Herein, *A*. *limacinum* OUC168 was engineered by gene knock-in of KR and DH using the 18S rDNA sequence as the homologous recombination site. Total fatty acid contents and the degree of unsaturation of total fatty acids increased after the *kr* or *dh* gene was knocked in. The cloning and functional study of the *pks3* gene of *A*. *limacinum* establishes a foundation for revealing the DHA synthetic pathway. Gene knock-in of the enzyme domain associated with PKS synthesis has the potential to provide effective recombinant strains with higher DHA content for industrial applications.

## Introduction

Omega-3 polyunsaturated fatty acids (ω-3 PUFAs), such as docosahexaenoic acid (DHA; C22:6, n-3) and eicosapentaenoic acid (EPA; C20:5, n-3), are beneficial to human health and have a variety of physiological effects on the human body [1, 2]. DHA is necessary for brain development in infants and can increase their intelligence [3]. In addition, DHA has a positive effect on treatments for cardiovascular diseases, Alzheimer’s disease [4], inflammation and autoimmune diseases [5, 6], as well as apoptosis of tumor cells and the prevention of cancers [7, 8]. The traditional source of DHA is fish oil, but the supply of high-quality fish oil is gradually decreasing because of seawater pollution and reduced catch [9]. Moreover, the terrible smell and taste of fish oil and the high extraction costs make it difficult to employ widely [10]. These problems have inspired people to seek new sources of DHA.

*Aurantiochytrium limacinum* is a species of unicellular marine fungi that is rich in lipid and polyunsaturated fatty acids (PUFAs). Total fatty acid contents reaches 50% of the dry weight of cells, particularly DHA, which exceeds 50% of total fatty acids [11]. *A*. *limacinum* synthesizes PUFAs by a specialized polyketide synthase (PKS) pathway [12], which synthesizes the PUFAs directly from acetyl-CoA and malonyl-CoA substrates.

In addition to *A*. *limacinum*, many bacteria synthesize PUFAs via the PKS pathway, including *Moritella marina* and *Shewanella pneumatophori* [13, 14]. In particular, different PUFA synthases contain similar enzyme domains. Metz et al. (2001) reported that *Shewanella* SCRC2738 has five open reading frames (ORFs) and at least 11 recognized enzyme functional domains in the frame. Three of these functional domains are related to fatty acid synthases (FAS) and the remaining eight functional domains are closely related to polyketide synthases (PKS). In 2001, Metz et al. first discovered three gene clusters from *Schizochytrium* (now called *Aurantiochytrium*), which encode proteins with highly similar PKS domains as those of *Shewanella* [15]. Therefore *A*. *limacinum* was considered to synthesize DHA through the PKS pathway. Until now, the specific function of these enzyme domains in *A*. *limacinum* has been equivocal.

In this study, the full-length *pks3* gene sequence (one of three *pks* genes) of *A*. *limacinum* was obtained by polymerase chain reaction amplification and Genome Walking technology. Based on the amino acid sequence analysis deduced from the *pks3* gene, the 3-ketoacyl-ACP reductase (KR) and the dehydratase (DH) enzyme domains were discovered. The predicted function of KR in the PKS pathway is to catalyze the hydroreduction of 3-ketoacyl-ACP to 3-hydroxyacyl-ACP. The predicted function of DH in the PKS pathway is to catalyze the introduction of a double bond to enoyl-ACP after dehydration of 3-hydroxyacyl-ACP [16]. However, the function of these two enzyme domains in *A*. *limacinum* has not been reported. The development of genetic transformation technology in *A*. *limacinum* has laid foundation for studying the gene function. 18S rDNA sequence as the homologous recombination site and PGK promoter and CYC1 terminator from *Saccharomyces cerevisia* as promoter and terminator were successfully applied to *A*. *limacinum* to express EGFP [17]. In this research, the *kr* and *dh* genes were knocked in *A*. *limacinum* to create the KR and DH overexpressing strains (*A*. *limacinum* KR and *A*. *limacinum* DH) respectively. Cell growth, gene transcription levels, and fatty acid composition were compared between *A*. *limacinum* OUC168, *A*. *limacinum* KR, and *A*. *limacinum* DH. Gene knock-in of the enzyme domains associated with PKS synthesis has the potential to provide effective recombinant strains for industrial applications.

## Materials and methods

### Strains, plasmids and mediums

*A*. *limacinum* OUC168 strain was preserved in our laboratory and used as the host for the transformation experiment.

Plasmid pTEF1/Zeo(*Ble*^*r*^ and *Amp*^*r*^)and plasmid pACYCDuet-1(*Cm*^*r*^) were purchased from Novagen (USA).

Solid medium [6% (w/v) glucose, 2% (w/v) yeast extract and 2% (w/v) Agar] with a salinity equivalent to 50% that of seawater was used for the conservation and selection of *A*. *limacinum* transformants at 23°C. Liquid medium [7% (w/v) glucose, 2% (w/v) yeast extract and 2% (w/v) sodium glutamate] with a salinity equivalent to 50% that of seawater was used for the propagation of *A*. *limacinum* [17].

### Cloning of full length of *pks3* gene

According to the known sequences obtained in our previous work, the primers (SP1,SP2,SP3) for cloning the 5 'end unknown sequences were designed (Table 1). The *pks3* 5'- unknown sequences were obtained using Genome Walking technology.

**Table 1:** Primers used in this study(underlined in the primer sequences were restriction enzyme sites).

Primers	Sequences	Product	Restriction enzyme sites
SP1	5'-GCGGACGCTA GGGTCGGTAG AGAAC-3'	*pks3* 5'-end unknown sequences	
SP2	5'-CGGGGAAGGA GTCAAGGCGC CACT-3'		
SP3	5'-CTTGAGGAGG GTGACGGTTT GGTTTGC-3'		
pks3F	5'-GGAATTCCATATGGCTCAACGTGAGAACCGTCTCGAG-3'	*pks3*	*Nde* I
pks3R	5'-ATAAGAATGCGGCCGCGAAGGCAAGGGAGTCAGAAGAGGTGA-3'		*Not* I
KR F	5'-CGCGGATCCATGAAGGCTTCCCTCTGCAC-3'	*kr*	*Bam*H I
KR R	5'-GCTCTAGACTATTAGCCAACAAGGATTTCAGC-3'		*Xba* I
DH F	5'-CGCGGATCCATGGGCAGTGAGCCCGTTGT-3'	*dh*	*Bam*H I
DH R	5'-GCTCTAGACTATTAAAGACCCTGGAAGGCAG-3'		*Xba* I
P_PGK_F	5'-CCGGAATTCTCTAACTGATCTATCCAAAAC-3'	*P*_*PGK*_	*Eco*R I
P_PGK_R	5'-CGCGGATCCATATTTGTTGTAAAAAGTAGATAATTAC-3'		*Bam*H I
T_CYC_F	5'-GCTCTAGACACGTCCGACGGCGGC-3'	*T*_*CYC*_	*Xba* I
T_CYC_R	5'-AAAACTGCAGAGCTTGCAAATTAAAGCCTTCGAGCG-3'		*Pst* I
18S+F	5'-GCGGGGCCCGTAGTGTACTGGACTACGGTG-3'	18S+	*Sma* I
18S+R	5'-CGAGCTCGCTTGGTAAATGCCTTCGCTC-3'		*Sac* I
18S-F	5'-AAAACTGCAGCTAGACCGTAAACGATGCCG-3'	18S-	*Pst* I
18S-R	5'-CCCAAGCTTGGACCGTTCAATCGGTAGGT-3		*Hin*d Ⅲ
Cmr F	5'-CGCGGATCCATGGAGAAAAAAATCACTGGATAT-3'	*Cm*^*r*^	*Bam*H I
Cmr R	5'-GCTCTAGATTACGCCCCGCCCTG-3'		*Xba* I

Subsequently, the primer pair *pks3* F/R(Table 1)were designed to amplify full length of the *pks3* gene according to the preliminary work of our laboratory and the sequence of the *A*. *limacinum pks3* gene that had been cloned in this research. Then the full length of *pks3* gene (GenBank accession number MH636606) was amplified to verify the sequence.

### Bioinformatics analysis of *pks3* gene

The *pks3* gene sequence was analyzed by DNAMAN (Lynnon Corporation, Quebec, Canada) and the BLAST algorithm [18] at the National Center for Biotechnology Information (NCBI) web site (http://www.ncbi.nlm.nih.gov/blast), and the amino acid sequence was analyzed by DNAMAN and the Expert Protein Analysis System (http://www.expasy.org) [19]. Secondary structure was predicted by NPS@: GOR4 secondary structure prediction [20]. The homology of amino acid sequences was analyzed by BLASTP software. BLASTP software was used to analyze the homology of amino acid sequences [21].The phylogenetic tree was constructed by neighbor-joining algorithms [22] of MEGA7 [23].

### Construction of gene knock-in recombinant vectors

Plasmid pTEF1 / Zeo was used as the original vector. The antibiotic Zeocin resistance gene (*Ble*^*r*^) was the screening marker gene. In addition, the *Cm*^*r*^ gene was newly introduced into the gene knock-in vector to make the gene knock-in transformants have the dual resistance of *Ble*^*r*^ and *Cm*^*r*^.

The gene sequences of complete KR enzyme domain (KR) and the DH enzyme domain (DH) were cloned into the pTEF1 / Zeo vector respectively. *PGK* promoter (P_PGK_) and *CYC1* terminator(T_CYC_)cloned from *Saccharomyces cerevisiae* were used as promoter and terminator for KR and DH respectively. 18Sr DNA upstream fragment (18S+) and 18Sr DNA downstream fragment (18S-) of *A*. *limacinum* were ligated to the both sides of KR or DH expression cassette, which were used as the homologous recombination sites. The primers (Table 1) for each fragment were designed with restriction enzyme sites and protective bases.

### Electrotransformation

Electrotransformation was used to transform the recombinant vector into *A*. *limacinum* following the reported protocols [24, 25]. 1.8 kV/cm, 200 Ω and 50 μF were the most suitable parameters for electrotransformation. After electrotransformation, the solution was recovered in 1 ml liquid medium without antibiotic and cultured at 28°C, 180 rpm/min, for 1 h.

### Screening of transformants

*A*. *limacinum* was found to be sensitive to chloramphenicol and zeocin [26]. Chloramphenicol(25.5–68 mg/L)or zeocin (2.5–4.0 mg/L) could restrain the growth of *A*. *limacinum*. Thence, chloramph*enicol* of 100 mg/L and zeocin of 5 mg/L were used for screening of gene knock-in transformants.

### Southern blotting

Southern blotting was used to detect whether the gene was knocked-in following the procedures described by Sun et al.[17]. The Yeast DNAiso Kit (Takara, Japan) was used to extract the genome DNA from *A*. *limacinum* OUC168 and the transformants respectively. One group of restriction enzymes (*Bam*HI/*Xba*I) was used to digest the DNA samples and the resultant DNA fragments were separated on 1% agarose gel and transferred to a nylon membrane (0.22 μm, Pall, USA). The DNA fragments of *kr* and *dh*, used as the probe respectively, were amplified from *A*. *limacinum* OUC168 with primers KR F/R and DH F/R separately and labeled with DIG. Probe detection in the Southern blotting was performed using a DIG High Prime DNA Labeling and Detection Starter Kit I (for color detection with NBT/BCIP) (Roche, USA).

### Real-time fluorescence quantitative PCR (qRT-PCR) analysis of the transformants

*A*. *limacinum* OUC168 and the transformants were cultured in 250ml liquid medium at 23°C for 5 days. All the samples collected on first, second, third and fourth day at a confirmed time (13:00 o’clock) to eliminate the differences in gene expression levels due to circadian rhythms. RNA was extracted respectively using the Yeast RNAiso Kit (Takara, Japan).

Reverse transcription was performed to obtain cDNA using the RT reagent Kit and gDNA Eraser (Perfect Real Time) kit (Takara, Japan). Real-time PCR was performed with the obtained cDNA. The housekeeping gene 18Sr RNA of *A*. *limacinum* was used as a reference gene. The specific primer pairs (Table 2) were designed based on the sequences of two target genes (*kr*, *dh*) and the reference gene 18Sr RNA. Real-time fluorescence quantitative PCR (qRT-PCR) was performed on an ABI 7500 FAST real-time PCR platform (USA) using SYBR Green PCR kits (Takara, Japan) according to the manufacturer’s instructions.

**Table 2:** Primers used for real-time fluorescence quantitative PCR.

Primers	Sequences
*kr*-F	5'- ATGAAGGCTTCCCTCTGCAC-3'
*kr*-R	5'- CCTCACGAGCAATATCAATACC-3'
*dh*-F	5'- GATCAGCGCTGCATCCAAC-3'
*dh*-R	5'- CACCCTTGAAGAGCTGAGCA-3'
18S rRNA F	5'- CTCAAAGATTAAGCCATGCATGTG-3'
18S rRNA R	5'- CATACAGGGCTCTTACAGCAT-3'

The data were processed by Microsoft Excel. The relative quantities of gene transcripts for the samples were analyzed by the 2^−ΔΔCt^ method [27].

### Biomass determination, total fatty acids extraction and fatty acid composition analysis

Cells were collected by centrifugation when *A*. *limacinum* OUC168 and transformants grew to logarithmic phase. The collected cells were washed twice with distilled water and freeze-dried 24 hours to obtain dry powder. The weight of the dry cells was weighed by a balance. The following formula was used to calculate biomass.

Biomass=celldryweight(g)/culturevolume(L)

The total fatty acids was extracted from the dry cells at room temperature using a combination of chloroform and methanol (2:1, v/v) following the procedures described by Song et al. [28]. The following formula was used to calculate total fatty acids content.

Totalfattyacidscontent=(weightoflipids(g)/celldryweight(g))*100%

The extracted fatty acids were converted to fatty acid methyl esters (FAMEs) by incubating the lipids in the presence of 2% (vol/vol) sulfuric acid in methanol at 85°C for 2.5 h and FAMEs were extracted using hexane to conduct gas chromatography following the method describe by Cheng et al. [29]. Fatty acid gas chromatography analysis was performed using an Agilent Technologies 7890B GC system (USA).The FAMEs were separated by a HP-INNOWAX (30 m × 0.25 mm i.d., 0.25 μm film thickness) capillary column. The oven temperature was initially set at 100°C for 1 min, then increased at 15°C /min up to 250°C and then preserved at 250°C for 5 min. The split ratio was 1:19 and the carrier gas was nitrogen. A flame ionization detector (FID) was used to carry out Peak detection and the temperature of the flame ionization port and injection port was 280°C, and the injection volume was 1 μL. The types of fatty acids were identified by mass spectrometry (Agilent 5975C, USA).

## Results

### Cloning and analysis of the *A*. *limacinum pks3* gene

**Fig 1:**
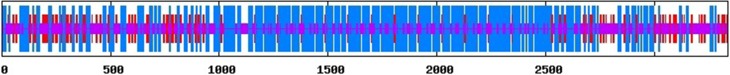
Secondary structure of polyketide synthase 3 in *A*. *limacinum*. Note: The longest blue stub show Alpha helix, the longer red stub show Extended strand and the shortest orange show Random coil.

The *A*. *limacinum pks3* gene contained 10,020 nucleotides with 51.2% GC, including an ORF with the initiation codon ATG at position 1 and the termination codon TAA at position 10020. No intron existed in the *A*. *limacinum pks3* gene, and the ORF encoded a protein of 3,339 amino acids (448 acidic amino acids and 355 basic amino acids) as deduced by DNAMAN. The putative molecular weight was about 351.5 kDa, and the theoretical isoelectric point was 4.89. The secondary structure of PKS3 included alpha helix (51.72%), extended strand (10.75%), and random coil (37.53%) regions as predicted by GOR4 analysis software (Fig 1).

**Fig 2:**
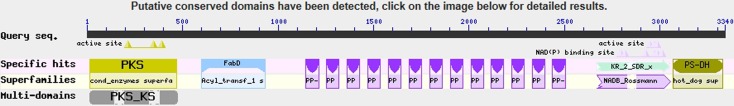
Conservative domains of amino acids of PKS3, including 3-ketoacyl synthetase enzyme domain (KS), acyltransferase enzyme domain (FabD), 3-ketoacyl-ACP reductase enzyme domain (KR), dehydratase enzyme domain (DH) and thirteen phosphopantetheine-binding sites (pp-binding site).

The conservative domains of the PKS3 amino acid sequence were analyzed by Blastp alignment (Fig 2). Thirteen phosphopantetheine-binding sites and four active enzyme domains, including the 3-ketoacyl synthetase enzyme domain, the acyltransferase enzyme domain, the KR, and the DH were found in the *A*. *limacinum* OUC168 PKS3.

**Fig 3:**
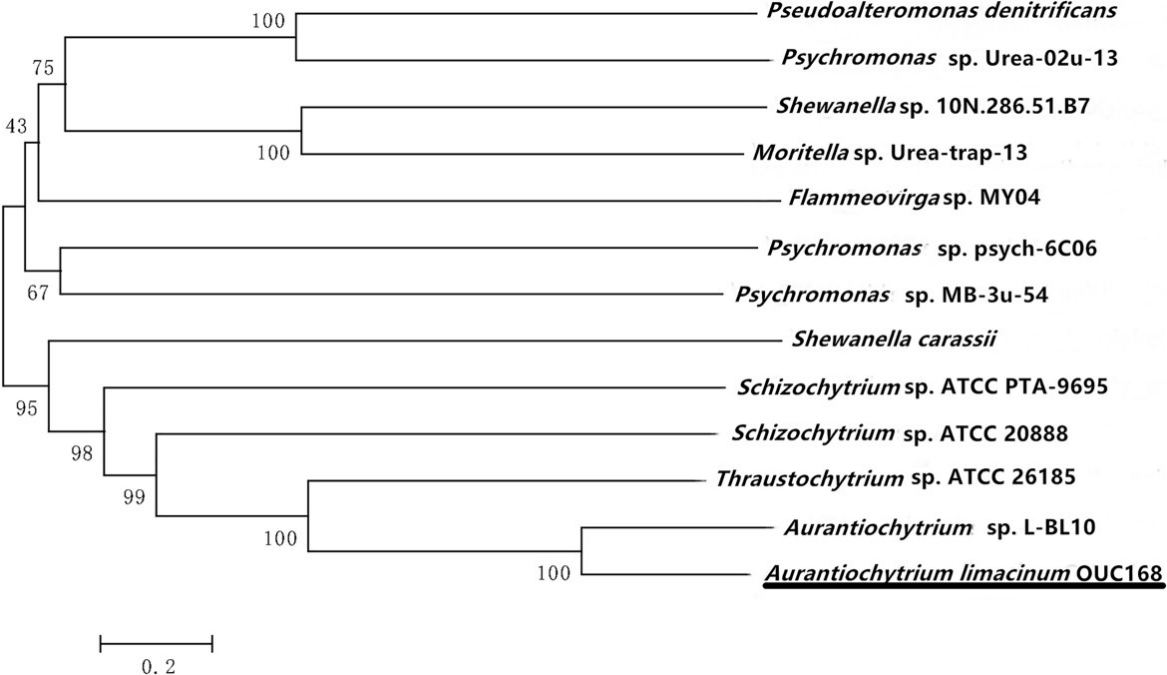
Phylogenetic tree of polyketide synthase in *Aurantiochytrium*. Numbers at the nodes represent the bootstrap values. The evolutionary distance between the groups is indicated by the scale (0.2 = 20% differences). The amino acid sequences taken from GenBank are as follows: *Aurantiochytrium limacinum* OUC168 (GenBank accession number MH636606), *Aurantiochytrium* sp. L-BL10 (AIJ29322.1), *Schizochytrium* sp. ATCC 20888 (AAK72879.2), *Thraustochytrium* sp. ATCC 26185 (AOG21004.1), *Schizochytrium* sp. ATCC PTA-9695 (APQ31260.1), *Shewanella carassii* (WP_100141973.1), *Psychromonas* sp. MB-3u-54 (WP_101041216.1), *Psychromonas* sp. psych-6C06 (WP_101109015.1), *Flammeovirga* sp. MY04 (WP_066209668.1), *Moritella* sp. Urea-trap-13 (WP_101062627.1), *Shewanella* sp. 10N.286.51.B7 (WP_102527861.1), *Psychromonas* sp. Urea-02u-13 (WP_101082395.1), and *Pseudoalteromonas denitrificans* (WP_091989355.1).

The PKS phylogenetic tree (Fig 3) was constructed by MEGA 7 using the neighbor-joining (NJ) method [23]. The results showed that *A*. *limacinum* OUC168 was most closely clustered with *Aurantiochytrium* sp. L-BL10 (GenBank No. AIJ29322.1) with 99% support. It was also clustered with *Thraustochytrium* sp. ATCC 26185 (GenBank No. AOG21004.1) and *Shewanella carassli* (GenBank No. WP_100141973.1). Polyketide synthase seems to be conserved. However, polyketide synthase from similar species clustered in different branches. The reason may be that polyketide synthase is composed of multiple enzyme domains corresponding to multiple gene clusters. In addition, polyketone synthase synthesizes many metabolites in addition to DHA. The main function of PKS3 is to synthesize DHA in *Aurantiochytrium* and to synthesize other metabolites in other species. Different functions may result from different sequences.

### Construction of the gene knock-in vectors and electrotransformation

**Fig 4:**
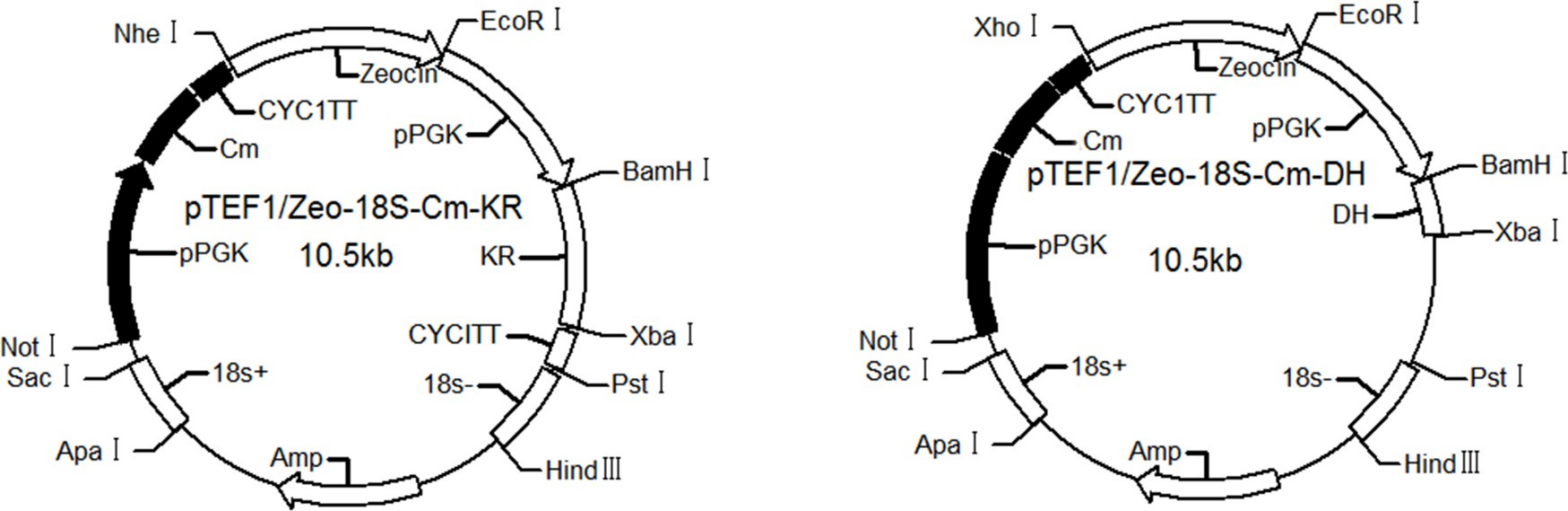
The structure of gene knock-in vectors.

The pTEF1/Zeo-18S-Cm-KR and pTEF1/Zeo-18S-Cm-DH plasmids (Fig 4) were the gene knock-in vector constructed with *kr* and *dh* as the target genes, respectively. The plasmids were trans*for*med into *A*. *limacinum* OUC168, and the transformants called *A*. *limacinum* KR and *A*. *limacinum* DH, respectively, were selected on solid medium containing zeocin and chloramphenicol.

### Hybridization detection in Southern blotting

**Fig 5:**
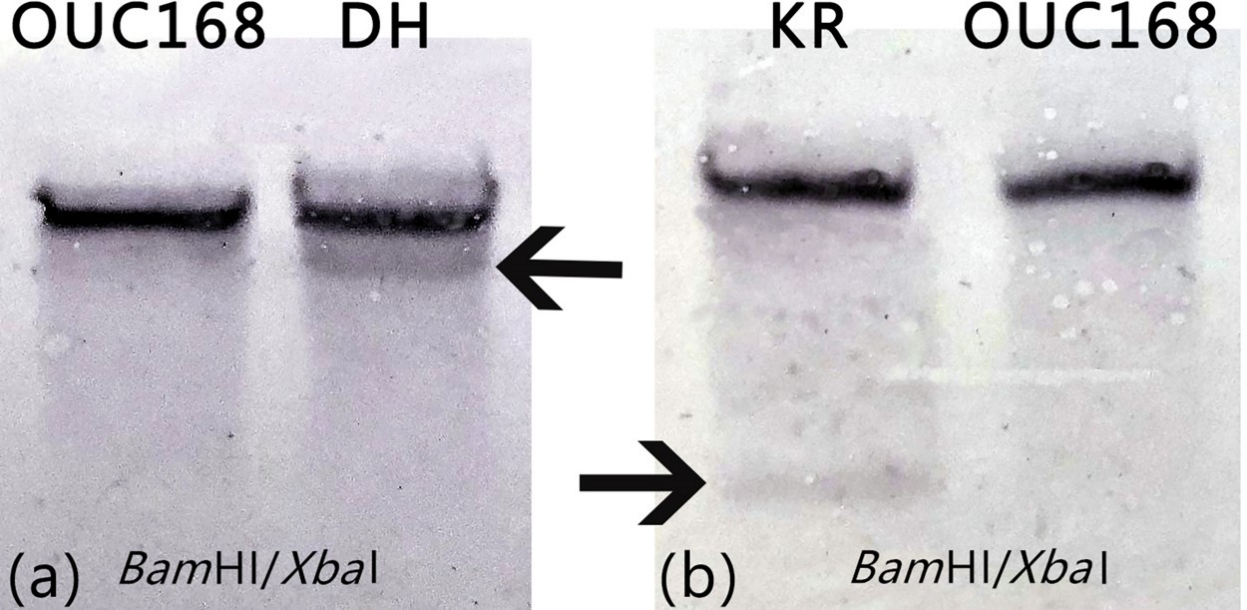
Southern blotting analysis of *kr* or *dh* in the genomic DNA of transformants. **(a)** The Southern blotting hybridization results for the *dh* gene **(b)** The Southern blotting hybridization results for the *kr* gene.

Genomic DNAs of all samples were individually digested by *Bam*HI/*Xba*I enzymes. The hybridization signals for Southern blotting were obtained using *kr* and *dh* as the probes, respectively. *A*. *limacinum* OUC168 had single hybridization signal. For *A*. *limacinum* KR and DH, two hybridization bands were found after digestion with *Bam*HI/*Xba*I, respectively (Fig 5). The extra band indicates that the target gene has been knocked into the *A*. *limacinum* OUC168.

### Transcriptional analysis of the transformants

**Fig 6:**
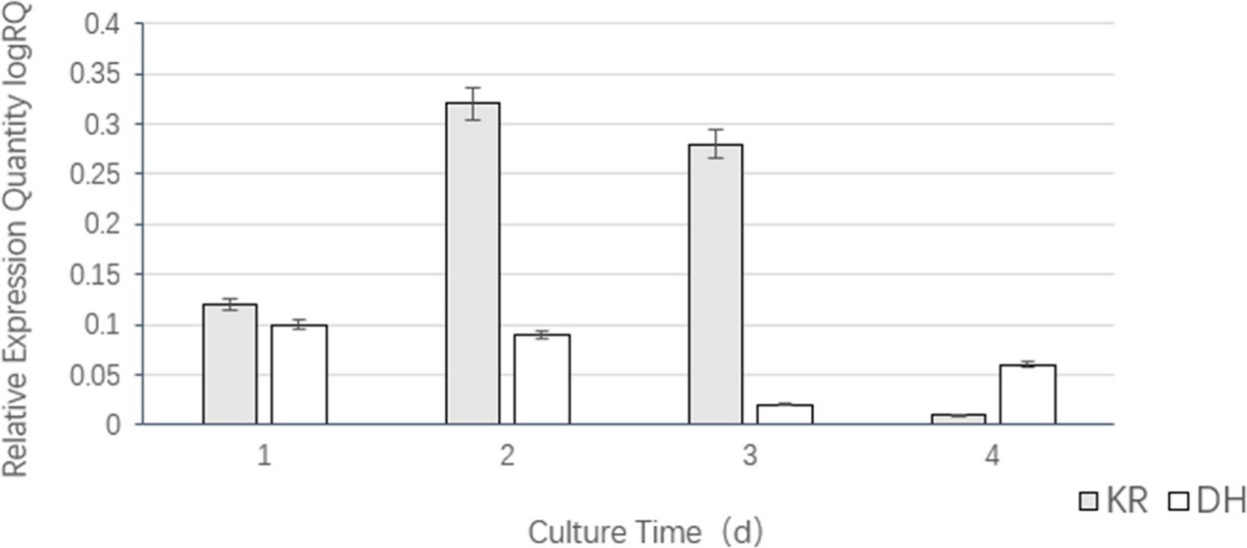
Relative transcription levels of *kr* and *dh* genes in transformed strains.

The gene transcription levels of the KR and DH transformed strains from the first day to the fourth day of fermentation are shown in Fig 6. The results show that the transcriptional levels of the *kr* and *dh* genes in the transformed strains (KR and DH) were higher than those of the untransformed strains, indicating increased transcription of the target genes. The transcription level of *dh* was higher on the first day, and subsequently tended to decrease and then increased, and was lowest on the third day. The *kr* transcriptional level was significantly higher than that of the *dh* transformed strain (P < 0.05), showing a trend of increasing first and then decreasing, and the transcription level increased sharply on the second day, but decreased to a level close to that of the untransformed strain on the fourth day.

### Biomass and total fatty acid content analyses

**Fig 7:**
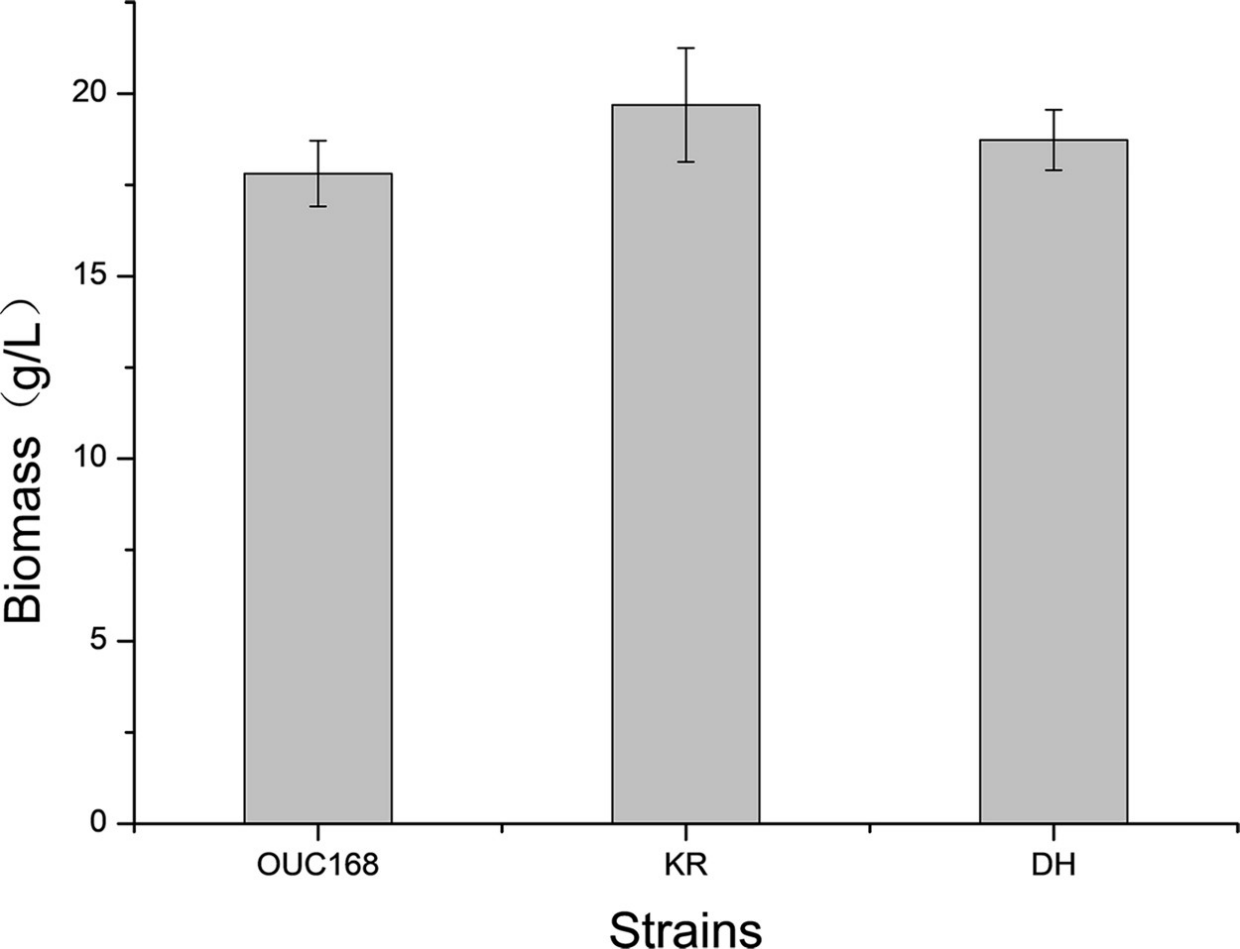
Biomass of strains *A*. *limacinum* KR, *A*. *limacinum* DH *and A*. *limacinum* OUC168. All data are expressed as means ± SD (n = 3).

The biomass of *A*. *limacinum* KR was highest (19.69 ± 2.36 g/L), followed by *A*. *limacinum* DH (18.73 ± 0.83 g/L) and *A*. *limacinum* OUC168 (17.81 ± 0.90 g/L) (Fig 7). The biomass of the transformed strains was slightly higher than that of the untransformed strains, but the difference was not significant (P > 0.05).

**Fig 8:**
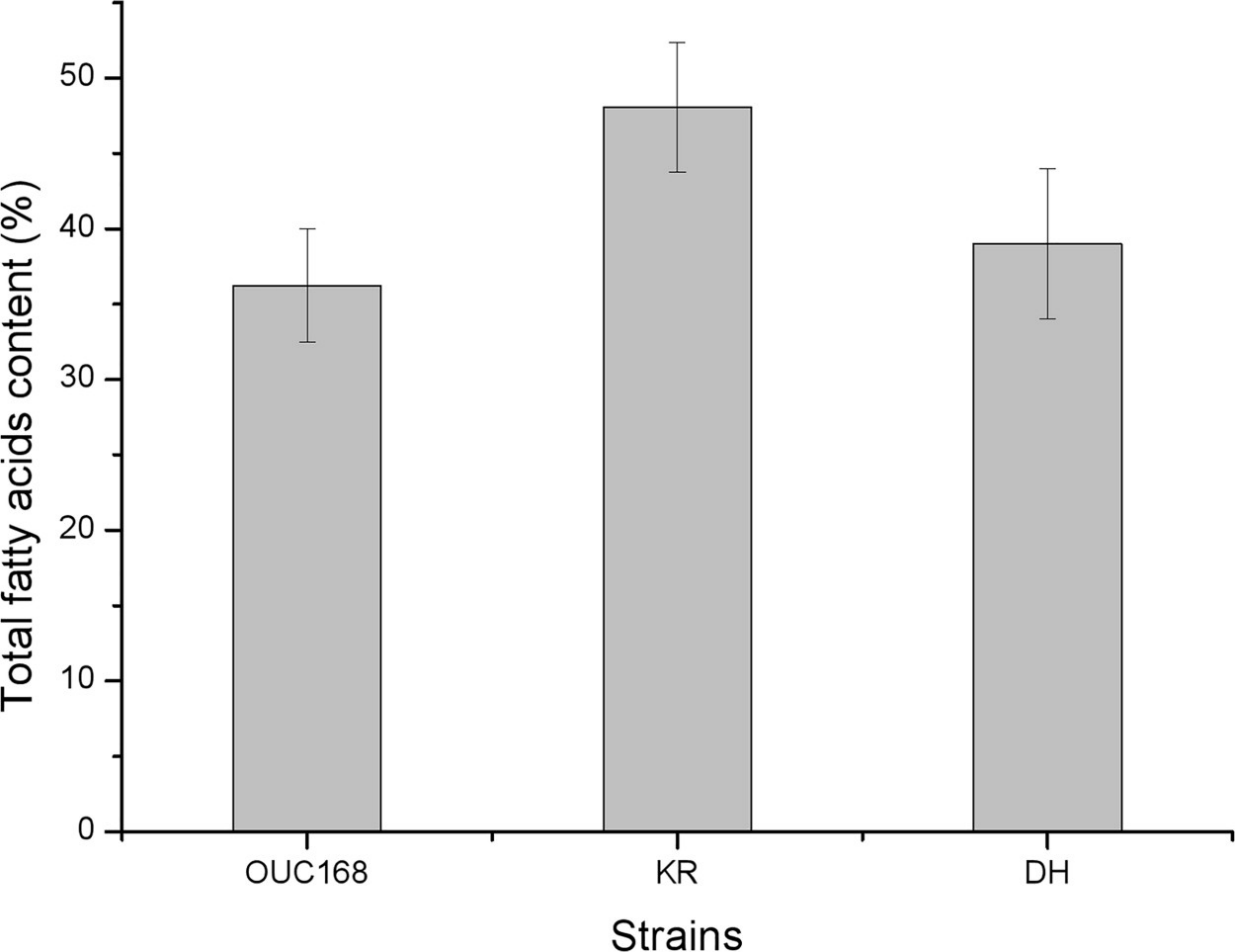
Total fatty acids content result of strains. All data are expressed as means ± SD (n = 3).

Total fatty acid contents of the strains are shown in Fig 8. Total fatty acid content of *A*. *limacinum* KR was the highest, reaching 48.07 ± 4.31%, followed by 39.00 ± 5.00% for *A*. *limacinum* DH and 36.24 ± 3.76% for *A*. *limacinum* OUC168. The total fatty acid content of the transformed strain *A*. *limacinum* KR was significantly higher than that of the untransformed strain (P < 0.05), but the difference between *A*. *limacinum* DH and the untransformed strain was not significant (P > 0.05).

The main fatty acids according to the gas chromatography-mass spectrometry analysis are listed in Table 3. The degree of unsaturation of total fatty acids increased after the *kr* or *dh* gene was knocked in, and specific fatty acids increased differently. After the *kr* gene was knocked in, DHA content increased by 4.33% compared to that in *A*. *limacinum* OUC168. After the *dh* gene was knocked in, C20:4 content increased significantly by 96.03%, and docosapentaenoic acid (DPA) content increased by 4.03%.

**Table 3:** Fatty acid composition of *A*. *limacinum* OUC168, KR and DH. All data are expressed as means ± SD (n = 3).

Fatty acids	Fatty acid composition(% of total fatty acids)		
	OUC168	KR	DH
C15:0	12.31±0.15	12.27±0.12	10.77±0.13
C16:0	16.38±0.11	15.05±0.09	15.45±0.14
C17:0	7.17±0.07	6.99±0.05	6.49±0.03
C20:4	1.01±0.02	0.89±0.03	1.98±0.07
DPA	10.95±0.04	10.98±0.06	11.41±0.03
DHA	45.45±0.11	47.42±0.17	45.07±0.12

## Discussion

*A*. *limacinum* is a high quality strain for producing DHA [30, 31] and is widely used for that purpose [32]. Increasing DHA content has always been a hot issue [32]. Optimizing the fermentation conditions improves DHA production to a certain extent, but the effect is limited [33]. It is possible to improve the synthetic ability of DHA through genetic modifications with the rapid development of modern biotechnology. However, the precondition of the genetic modification is to clarify the fatty acid synthetic pathway in *Aurantiochytrium*. Three gene clusters- *pks1*,*pks2* and *pks3* were reported to perform main function on synthesis of DHA [34]. In this study, the full-length *pks3* gene was cloned from *A*. *limacinum* OUC168, and the KR and DH enzyme domains were discovered in PKS3, which reportedly play important roles in PUFA biosynthesis. The KR and DH gene knock-in strains were constructed to further study the function of the KR and DH enzyme domains in *A*. *limacinum*. The effects of KR or DH on biomass, transcription levels, and fatty acid synthesis were systematically studied.

The predicted function of KR in the PKS pathway was to catalyze the hydroreduction of 3-ketoacyl-ACP to 3-hydroxyacyl-ACP and elongate acyl fatty acid precursors [35]. KR is highly conserved and ubiquitously expressed in all bacteria, and is the only known isozyme that catalyzes the essential keto reduction step in the FAS II elongation cycle [36]. One study showed that KR is essential for survival of some species, such as *Mycobacterium tuberculosis* [37]. The predicted function of DH in the PKS pathway was to catalyze the introduction of a double bond to enoyl-ACP after dehydration of 3-hydroxyacyl-ACP. According to one report, an *Escherichia coli* strain engineered to overexpress a fragment consisting of four DH domains from the PUFA synthase enzyme complex, and the results showed that the *E*. *coli* strain expressing the DH tetradomain fragment was capable of producing up to a five-fold increase in total fatty acids over the negative control strain lacking the recombinant enzyme [38]. Cao et al. engineered an efficient producer of unsaturated fatty acids by overexpressing two genes (*fabA* and *fabB*) associated with unsaturated fatty acid synthesis in *E*. *coli* [39]. Thus, these studies predicted that the KR and DH enzyme domains are important for synthesizing fatty acids. The KR and DH enzyme domains catalyze the reduction of carbon chains and increase the degree of carbon chain unsaturation. In this study, the degree of unsaturation of total fatty acids increased after the *kr* or *dh* gene was knocked in, which further indicates that KR and DH play an important roles in the dehydration and reduction of fatty acids.

Constructing overexpressing transformants increases synthesis of the product [40]. An effective way to enhance the synthesis of products is to increase expression of the corresponding genes [41]. Glucose-6-phosphate dehydrogenase has been overexpressed in *A*. *limacinum*, which changes the fatty acid profile and enhances the proportion of PUFAs among lipids [33]. Furthermore, *A*. *limacinum* was engineered via gene deletion of the acyltransferase (AT) domain and replacement of the native AT with its homologue, the Shew-AT domain from *Shewanella* sp., with 3.7 times more EPA [12]. According to the results, the transcription levels of the overexpressing strains were significantly higher than that of *A*. *limacinum* OUC168. Furthermore, total fatty acid content and specific unsaturated fatty acid contents of *A*. *limacinum* KR and *A*. *limacinum* DH increased significantly. The DHA content of the KR overexpressing strain increased by 4.33% compared to *A*. *limacinum* OUC168. The C20:4 content of the DH overexpressing strain increased significantly by 96.03%, and the DPA content in the DH overexpressing strain increased by 4.03%. All of these results demonstrate that overexpressing KR and DH is an effective way to promote the synthesis of PUFAs in *A*. *limacinum*.

In this study, *A*. *limacinum* OUC168 was engineered by gene knock-in of KR and DH using 18S rDNA sequences as the homologous recombination sites. The KR and DH gene knock-in strains were obtained using an electroporation technique. In recent years, genetic engineering methods have been successfully applied to *A*. *limacinum* to increase DHA content and to study the function of polyketide synthase-related enzyme domains. A zeocin resistance gene has been introduced into *A*. *limacinum* using particle bombardment technology [42]. The Cre/loxP site-specific recombination system has been applied to *A*. *limacinum* to obtain a transformant without the antibiotic resistance marker gene using 18Sr DNA sequences as the homologous recombination sites [17]. These studies have provided us with numerous genetic transformation methods. In this study, zeocin and chloramphenicol were used to screen the double-resistant strains, which effectively avoided a false positive.

This study showed that the KR and DH enzyme domains are important for synthesizing DHA. This research also provides new strains for industrial production of DHA from *A*. *limacinum*.
